# Prevalence, risk factors, and psychosocial impacts of enuresis in Saudi primary school-aged children: a systematic review and meta-analysis

**DOI:** 10.11604/pamj.2026.53.46.50243

**Published:** 2026-01-29

**Authors:** Khaled Abdulwahab Aldhabaan, Raed Abdullah Almuhsin, Nora Khaled Al Shehri, Abdullah Ali Almazni, Ahmed Ibrahim Almania, Khalid Abdullah Alasmari, Waleed Ibrahim Alshardi, Abdulaziz Saeed Alserhani, Saad Dhafer Alshahrani

**Affiliations:** 1 Khamis Mushayt Maternity and Children Hospital, Asir Health Cluster, Abha, Saudi Arabia,; 2 Asir Central Hospital, Asir Health Cluster, Abha, Saudi Arabia,; 3 Ouhd Rafidah General Hospital, Asir Health Cluster, Abha, Saudi Arabia,; 4 College of Medicine, King Khalid University, Abha, Saudi Arabia,; 5 Surgery Department, College of Medicine, University of Bisha, Bisha, Saudi Arabia

**Keywords:** Enuresis, prevalence, risk factors, Saudi children

## Abstract

Enuresis is a common yet often under-recognized childhood condition associated with substantial emotional and psychosocial consequences. Although multiple studies from Saudi Arabia have examined its prevalence and correlates, the available evidence remains fragmented and methodologically heterogeneous. This systematic review and meta-analysis aimed to synthesize existing data on the prevalence of nocturnal enuresis among Saudi primary school-aged children and to summarize associated risk factors and psychosocial impacts. A comprehensive search of PubMed, Scopus, Web of Science, and Google Scholar identified observational studies published up to October 2025 involving children aged 5-16 years. Seven studies comprising 5,369 participants met the inclusion criteria and were assessed for methodological quality using the Newcastle-Ottawa Scale. Pooled prevalence was estimated using a random-effects model. The overall pooled prevalence of nocturnal enuresis was 22% (95% CI: 17-27%), with substantial heterogeneity (I^2^=99.4%). Sensitivity analysis excluding a methodological outlier reduced the pooled prevalence to 14.2% (95% CI: 8.4-23.0%), indicating that prevalence estimates should be interpreted as a range rather than a single definitive value. Primary nocturnal enuresis accounted for approximately 71% of reported cases. Consistently reported risk factors included positive family history, younger age, psychosocial stressors, sleep disturbances, obesity, and recurrent urinary tract infections. Enuresis was also associated with significant psychosocial consequences, including embarrassment, reduced self-esteem, emotional distress, and impaired school performance. Overall, nocturnal enuresis remains a prevalent and under-addressed condition among Saudi primary school-aged children. The findings underscore the need for improved parental and clinical awareness, early identification, and supportive, non-punitive management approaches. Integrating clinical care with parental education and school-based health initiatives may help reduce stigma and mitigate the psychosocial burden of enuresis in this population.

## Introduction

Enuresis, often referred to as bedwetting, is a common elimination disorder in childhood that involves involuntary urination during sleep in children aged five years or older without any identifiable organic cause [1]. According to the Diagnostic and Statistical Manual of Mental Disorders, Fifth Edition (DSM-5), the condition is diagnosed when episodes occur at least twice per week for three consecutive months or when they lead to significant emotional distress [2]. Two main types are described: monosymptomatic nocturnal enuresis (MNE), which occurs in the absence of daytime urinary symptoms, and non-monosymptomatic nocturnal enuresis (NMNE), where symptoms such as urgency or daytime incontinence are present [3]. Monosymptomatic nocturnal enuresis (MNE) may be primary, when the child has never maintained a dry period at night, or secondary, when bedwetting reappears after at least six months of dryness [1].

Globally, nocturnal enuresis is one of the most frequent developmental disorders in childhood. It affects about 15 percent of children aged five years and around 10 percent of those aged seven years, with spontaneous resolution occurring in approximately 15 percent of cases annually [4]. However, a small proportion, estimated at one to two percent, continue to experience symptoms into adulthood. Boys are more frequently affected than girls, and family history is a well-established risk factor [4]. The likelihood of enuresis increases to about 40 percent when one parent is affected and exceeds 70 percent when both parents have the condition [5]. Physiological mechanisms that contribute to enuresis include delayed bladder control, nocturnal polyuria due to low antidiuretic hormone secretion, detrusor muscle overactivity, small functional bladder capacity, and difficulty arousing from sleep [6]. Environmental and psychosocial stressors, including parental separation or family conflict, are more commonly associated with secondary enuresis [7].

The condition also carries significant psychosocial consequences. Children often report embarrassment, loss of confidence, social withdrawal, and reduced academic performance [7]. Negative parental responses, such as scolding or punishment, may intensify the child´s emotional distress [8]. Research indicates that a notable proportion of children with enuresis also exhibit behavioral or emotional difficulties such as anxiety, depression, or attention-deficit symptoms [9]. In Saudi Arabia, epidemiological studies have reported markedly heterogeneous prevalence estimates, ranging from approximately 7% to over 70%, depending on geographic region, age range, sampling strategy, and diagnostic criteria [10,11].

The most recent national evidence suggests a pooled prevalence of roughly 25 percent [12]. Familial history, stressful events, and sleep-related problems are consistent risk factors, while embarrassment and social stigma remain common psychosocial effects. Despite the availability of effective management strategies, many children remain untreated. Therefore, a systematic review and meta-analysis focusing on Saudi primary school-aged children is essential to synthesize the available evidence, clarify the range and drivers of reported prevalence, and integrate data on associated risk factors and psychosocial consequences. Such an approach may provide a more nuanced understanding of nocturnal enuresis in the Saudi context and help inform culturally appropriate clinical and public health interventions.

## Methods

**Study design:** this systematic review and meta-analysis aimed to evaluate the prevalence, risk factors, and psychosocial impacts of enuresis in Saudi primary school-aged children. The study was conducted in accordance with the Preferred Reporting Items for Systematic Reviews and Meta-Analyses (PRISMA) 2020 statement and the Cochrane Handbook for Systematic Reviews of Interventions [13,14]. The review protocol was not prospectively registered in PROSPERO or any other registry. The study was guided by the PICO framework as follows: population (P): children aged 5-12 years residing in Saudi Arabia; intervention/exposure (I): presence of enuresis; comparison (C): children without enuresis or varying severity/frequency of enuresis; outcomes (O): prevalence, associated risk factors (e.g., parental history, psychosocial stressors, sleep disorders), psychosocial impacts, and management strategies.

**Search strategy:** a comprehensive and systematic literature search was conducted across multiple electronic databases, including PubMed (MEDLINE), Scopus, Web of Science, and Google Scholar, from their inception until October 2025. The search strategy was designed to capture all relevant studies. Both controlled vocabulary terms (e.g., MeSH) and free-text keywords were used, encompassing variations of the terms “nocturnal enuresis,” “enuresis,” “bedwetting,” “urinary incontinence,” “children,” and “Saudi Arabia”. These terms were combined using Boolean operators (AND/OR) to ensure a sensitive and comprehensive search. No restrictions were placed on publication year; however, searches were limited to human studies published in English. Additional manual searches were performed through reference lists of relevant articles and gray literature sources to identify any studies not captured in the database searches. For Google Scholar, the first five pages of search results were screened, as the platform´s algorithm prioritizes studies with the highest relevance to the search strategy in the initial results.

**Selection criteria:** studies were included if they met the following criteria: (1) conducted among children or adolescents residing in Saudi Arabia; (2) reported the prevalence, characteristics, associated factors, or management of enuresis; and (3) utilized an observational study design, such as cross-sectional, cohort, or case-control studies. Only studies published in English and involving human participants were considered.

Exclusion criteria included: (1) studies conducted outside Saudi Arabia; (2) studies focusing exclusively on adults or non-human populations; (3) case reports, reviews, editorials, conference abstracts, or letters to the editor without sufficient primary data; and (4) studies that did not clearly define or measure enuresis. When duplicate publications were identified, the most comprehensive or recent version of the study was included.

**Figure 1:**
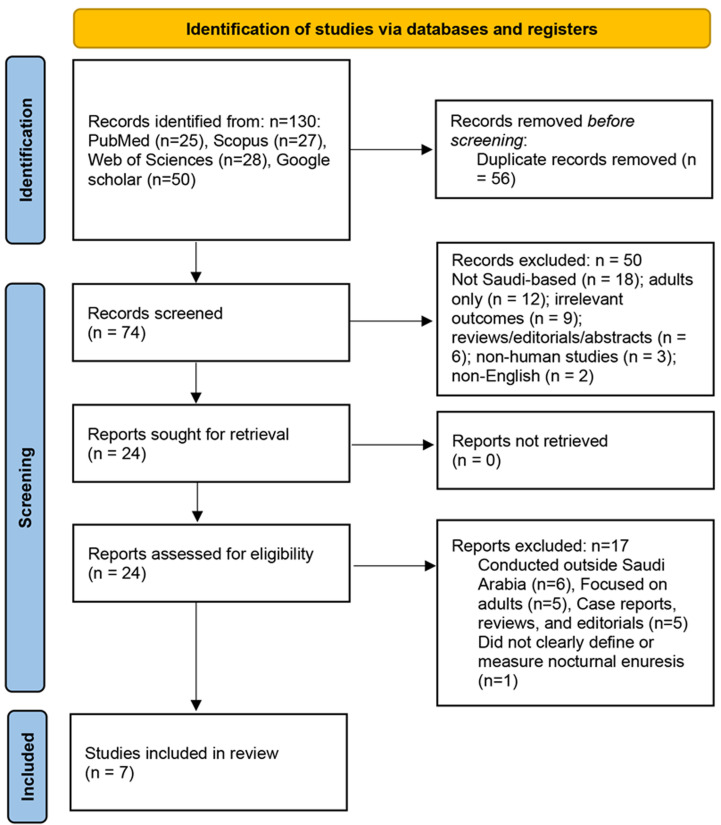
PRISMA flow diagram illustrating the selection process for studies included in the systematic review and meta-analysis of enuresis among Saudi primary school-aged children (the diagram shows the number of records identified, screened, excluded, and included at each stage)

**Study selection:** all identified records were imported into Rayyan [15] for duplicate removal. Title and abstract screening were performed independently and in duplicate, with the six reviewers divided into three pairs. Each record was screened by two reviewers working independently. Full texts of potentially eligible studies were then assessed independently and in duplicate by four reviewers, working in two reviewer pairs, against the predefined inclusion and exclusion criteria. Discrepancies were resolved through discussion or consultation with a senior reviewer. The study selection process is summarized in the PRISMA flow diagram (Figure 1).

**Data extraction:** data were extracted independently and in duplicate by four reviewers using a standardized Excel data extraction form. Extracted variables included study characteristics (author, year, region, design, sample size, age, sex distribution), prevalence and type of enuresis, associated risk factors, psychosocial impacts, and management strategies. When necessary, corresponding authors were contacted to clarify missing or unclear data. Extracted data were cross-verified to ensure accuracy.

**Quality assessment:** the methodological quality of included studies was assessed using the Newcastle-Ottawa Scale (NOS) for observational studies [16], which evaluates participant selection, comparability, and outcome ascertainment. Quality assessment was conducted independently and in duplicate by four reviewers, with each study assessed by two reviewers. Studies were categorized as high (7-9), moderate (4-6), or low quality (0-3). Any disagreements were resolved through discussion and, if required, consultation with a senior reviewer.

**Statistical analysis:** meta-analyses were conducted using R software (version 4.3.3) with the meta and metafor packages. Pooled prevalence estimates were calculated using random-effects models. Statistical heterogeneity was assessed using the I^2^ statistic and Cochran´s Q test. Formal assessment of publication bias was not performed, as the number of included studies (n=7) was below the minimum recommended threshold of 10 studies. Funnel plot-based methods and statistical tests for small-study effects, such as Egger´s regression test or Begg´s rank correlation test, are known to have low statistical power and may yield misleading results when applied to fewer than 10 studies [17,18]. Therefore, in accordance with methodological guidance, publication bias was not formally evaluated. Sensitivity analyses using a leave-one-out approach were performed to assess the robustness of pooled estimates and to explore the influence of individual studies on overall results. Statistical significance was set at p <0.05.

## Results

**Study selection:** the initial search across PubMed, Scopus, Web of Science, and Google Scholar yielded 130 records (Figure 1). After the removal of 56 duplicate records, 74 records were screened by title and abstract. Of these, 50 records were excluded for the following reasons: studies not conducted in Saudi Arabia (n = 18), studies involving adult populations only (n = 12), studies with irrelevant outcomes not related to nocturnal enuresis (n = 9), review articles, editorials, or conference abstracts (n = 6), non-human studies (n = 3), and non-English publications (n = 2). The full texts of 24 reports were then assessed for eligibility, of which 17 were excluded because they were conducted outside Saudi Arabia (n = 6), focused on adult populations (n = 5), were case reports, reviews, or editorials lacking primary data (n = 5), or did not clearly define or measure nocturnal enuresis (n = 1). Ultimately, seven studies met the inclusion criteria and were included in the systematic review and meta-analysis [10,11,19-23].

**Characteristics of the included studies:** Table 1 summarizes the characteristics of the included studies. All studies employed a cross-sectional design, with sample sizes ranging from 152 to 2,701 children, and participants aged 5-16 years. The male-to-female ratio varied across studies, with some studies including only male participants (Alnajjar *et al*.) [21]. The number of children diagnosed with nocturnal enuresis ranged from 13 (8.5%) to 386 (76.4%). Parental or sibling history of enuresis was reported in some studies, with the highest rates observed in Alhejji *et al*. [19] (parents: 44.4%; siblings: 83.3%).

**Table 1:** characteristics of included studies assessing enuresis among Saudi primary school-aged children, conducted between 2014 and 2023 across various regions of Saudi Arabia (N=5,369 participants)

Study	Study design	Settings	Population	Total sample size	Age characteristics	Gender (male/female)	Number of cases (%)	Parents suffering from NE N (%)	Sibling suffering from NE N (%)
Al-Zahrani *et al*. 2014	Cross-sectional (questionnaire through interview)	Taif city	Children aged 7-12 years	2701	NA	1501/1200	211 (7.8)	NA	NA
Alnajjar 2017	Cross-sectional (questionnaire)	Al-Eskan region, Makkah	Children aged 6-12 years	152	9.5 ± 1.8	152/0	13 (8.5)	NA	25 (16.45)
Shahin *et al*. 2017	Cross-sectional (online questionnaire)	Hail	Children aged 5-12 years	652	41.1% were 5-7 years	286/365	148 (22.6)	34 (5.21)	54 (8.28)
Alshahrani *et al*. 2018	Cross-sectional (online questionnaire)	Riyadh City	Children aged 5-12 years	352	43.5% were 5-6 years	39/23	65 (18.4)	27 (7.67)	NA
Sherah *et al*. 2019	Cross-sectional (questionnaire)	Jazan	Children aged 5-12 years	505	45% were 5-7 years	254/251	386 (76.4)	NA	NA
Alhejji *et al*. 2021	Cross-sectional	Jeddah	Primary school students (5-16 years)	321	9.9 ± 2.11 (for cases)	173/148	36 (11.2)	16 (44.4)	30 (83.3)
Alshehri *et al*. 2023	Cross-sectional (online questionnaire)	All Saudi Arabia regions	Children aged 6-12 years	686	36.7% were 6-7 years	351/335	153 (22.3)	NA	NA

NA: not reported; NE: nocturnal enuresis

**Figure 2:**
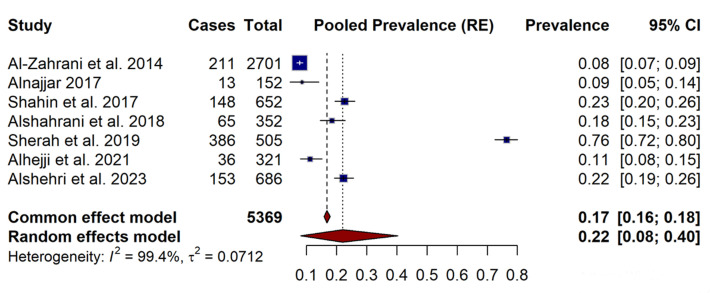
forest plot showing the pooled prevalence of enuresis among Saudi primary school-aged children, derived from seven included studies (N=5,369 participants) using a random-effects meta-analysis model (individual study estimates, and 95% confidence intervals are displayed alongside the overall pooled prevalence)

**Figure 3:**
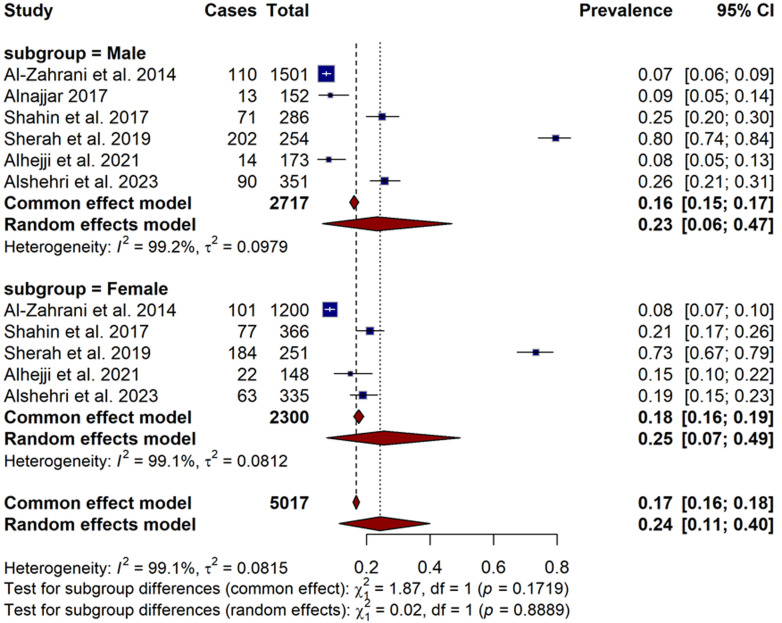
forest plot depicting the pooled prevalence of enuresis stratified by gender (male vs. female) among Saudi primary school-aged children, based on available subgroup data from included studies (the estimates were generated using a random-effects model)

**Prevalence of nocturnal enuresis:** the meta-analysis included seven studies with a total of 5,369 children. Reported prevalence rates varied widely from 8% (Al-Zahrani *et al*.) to 76% (Sherah *et al*.) (Figure 2) [10,11]. Due to extreme heterogeneity (I^2^= 99.4%), a random-effects model was used. The pooled prevalence of nocturnal enuresis across studies was 22% (95% CI: 8-40%). Subgroup analysis by gender (Figure 3) revealed a pooled prevalence of 23% (95% CI: 6-47%) in males (n = 2,717) and 25% (95% CI: 7-49%) in females (n = 2,300). Heterogeneity remained very high in both subgroups (I^2^= 99.2% for males, 99.1% for females), and no statistically significant difference between genders was observed (p = 0.89).

**Sensitivity analysis:** given the extreme heterogeneity observed in the primary meta-analysis, a leave-one-out sensitivity analysis was conducted by excluding the study by Sherah *et al*. which reported an unusually high prevalence of nocturnal enuresis. The reanalysis included six studies comprising 4,864 children, of whom 626 had nocturnal enuresis. Individual study prevalence estimates ranged from 7.8% to 22.7%. Using a random-effects model with logit transformation and Hartung-Knapp adjustment, the pooled prevalence of nocturnal enuresis decreased to 14.2% (95% CI: 8.4-23.0%). Although heterogeneity was modestly reduced, it remained substantial (I^2^= 97.1%, p < 0.0001), indicating considerable between-study variability beyond chance.

**Type of enuresis:** three studies (n = 435 cases) reported the proportion of primary enuresis among affected children. Individual proportions ranged from 53% to 85%, with substantial heterogeneity (I^2^= 87.6%). The pooled proportion of primary nocturnal enuresis was 71% (95% CI: 49-89%).

**Associated factors and psychosocial impacts:** the included studies reported a range of factors associated with nocturnal enuresis and its psychosocial consequences (Table 2). Familial history, including parental and sibling enuresis, emerged as a consistent risk factor, alongside psychosocial stressors, sleep-related issues such as deep sleep or snoring, lower parental education, recurrent urinary tract infections, stool incontinence, obesity, and aspects of family structure, including single-parent households or large family size. Nocturnal enuresis also had notable psychosocial impacts on affected children, with many experiencing embarrassment, frustration, or carelessness, while parental responses ranged from supportive counseling and education to blaming or punitive actions. In some studies, enuresis was associated with impaired school performance. Management practices varied widely, including the use of enuresis alarms, fluid restriction, medication, waking the child for voiding, and traditional or home-based strategies. However, a substantial proportion of children did not receive formal medical treatment.

**Table 2:** summary of enuresis characteristics, associated risk factors, psychosocial impacts, and management strategies reported in studies conducted among Saudi primary school-aged children between 2014 and 2023

Study	Enuresis characteristics (type and frequency)	Key associated factors (risk Factors)	Psychosocial impacts	Management strategies
Al-Zahrani *et al*. 2014	Prevalence is higher in girls (8.4%) than in boys (7.3%)	NA	NA	Methods used: Enuresis alarm (56.9%), water restriction (14.7%), medication (5.7%), awaking for voiding (5.7%), no consultation (17.1%)
Alnajjar 2017	Type: primary (84.6%), secondary (15.4%)	Significant association with: recurrent UTI, stool incontinence, parental consanguinity, low family income, low paternal education, living with a single parent, parental separation, and acute family problems	Significantly associated with parental separation and acute psychological/social family problems	NA
Shahin *et al*. 2017	Frequency: >3 times/week (30.7%), 3 times/week (18.4%), 1-2 times/week (16.3%)	Associated with: deep sleep (51.5% of cases), sleep disorders (e.g., somnambulism, 4.1%), parents being separate (20.2%), sleeping in a cold room, and watching horror cartoons	Child's reaction: embarrassment (59.8%), carelessness (26.5%). Mother's reaction: receptive (61.5%), nervous (29.1%), beating (4.8%)	27.0% visited a doctor. 40.0% used traditional methods
Alshahrani *et al*. 2018	Frequency: every week (56.5%), every night (38.7%); prevalence decreases with age	Associated with: stressful events (67.7% of cases), new child birth (22.6%), parents' divorce (6.5%), and family size >5; no relation found with parental education	Stressful events were a key factor	29% tried treatment; methods: fluid restriction and awaking (12%), medical consultation and fluid restriction (6.1%)
Sherah *et al*. 2019	Type: primary (52.6%); prevalence increased with age (highest at 9-12 years, 85.6%)	Significant association with: pinworm infestation, lower father's education, and not being normally breastfed	School performance: significantly associated with “good” (48.4%) vs. “excellent” performance; no significant link to stressful events or punishment.	77.4% received no proper treatment; 38.2% sought medical advice. Methods: Voiding before sleep (75.3%), fluid restriction (21.4%)
Alhejji *et al*. 2021	Type: primary (80%), secondary (20%); severity: severe (52.9%), moderate (39.0%)	Significant association with: parental history, sibling history, stressful events, fearing going to the bathroom alone, and having a working mother	Parental attitude: counseling/learning (57.1%), blaming (24.6%), hitting (14.3%)	19.4% consulted doctors; methods: leave to resolve (42.9%), medication (20.0%), awaking for voiding (20.0%), reduce water intake (17.1%).
Alshehri *et al*. 2023	Frequency: <2 times/week (63.4%), 2+ times/week (36.6%); prevalence is lower in 10+ age group	Significant association with: obesity (40.2% of obese children had NE), sleeping before 10 PM, snoring, difficulty breathing, and mouth breathing (sleep-disordered breathing symptoms)	NA	NA

NA: not reported

**Quality assessment of included studies:** the methodological quality of the included studies, assessed using the Newcastle-Ottawa Scale (NOS), is presented in Table 3. Total scores ranged from 6 to 8, with five studies rated as moderate quality and three studies as high quality. Selection scores were generally high (3/4), while comparability and outcome/exposure scores varied depending on the reporting of confounding factors and outcome ascertainment.

**Table 3:** quality assessment of the included studies using the Newcastle-Ottawa Scale (NOS), evaluating selection (maximum 4 points), comparability (maximum 2 points), and outcome/exposure assessment (maximum 3 points), for a total possible score of 9 points

Study	Selection (max: 4)	Comparability (max: 2)	Outcome/exposure (max: 3)	Total score (max: 9)	Quality rating
Al-Zahrani *et al*. 2014	3	1	2	6	Moderate
Alnajjar 2017	3	2	2	7	High
Shahin *et al*. 2017	3	1	2	6	Moderate
Alshahrani *et al*. 2018	3	1	2	6	Moderate
Sherah *et al*. 2019	3	1	2	6	Moderate
Alhejji *et al*. 2021	3	2	2	7	High
Alshehri *et al*. 2023	3	2	3	8	High

## Discussion

This systematic review and meta-analysis synthesized available data on the prevalence, associated risk factors, and psychosocial impacts of enuresis among Saudi primary school-aged children. The overall pooled prevalence of nocturnal enuresis was estimated at approximately 22%, suggesting that bedwetting affects a substantial proportion of school-aged children in Saudi Arabia. However, this estimate lies at the upper end of reported global prevalence rates and should be interpreted cautiously in light of the substantial heterogeneity observed across studies.

Importantly, sensitivity analysis excluding a methodological outlier with an exceptionally high reported prevalence resulted in a lower pooled estimate of approximately 14%, indicating that the summary prevalence is sensitive to individual study characteristics and should be viewed as a range rather than a single definitive value. This finding reinforces the importance of contextualizing pooled prevalence estimates in epidemiological meta-analyses of developmental conditions, particularly when diagnostic criteria and study designs vary widely [24].

Primary nocturnal enuresis accounted for approximately 71% of all cases, consistent with the predominance of the primary type worldwide. Familial aggregation was evident, as both parental and sibling histories were repeatedly identified as significant risk factors. Other contributing factors included psychosocial stress, sleep-related problems, low parental education, urinary tract infection, obesity, and larger family size. Psychosocial outcomes, such as embarrassment, frustration, and impaired school performance, were frequently reported, highlighting the emotional burden and stigma surrounding the condition.

The observed prevalence in this study exceeds many reports from other regions. A recent global meta-analysis involving 39 countries estimated a pooled prevalence of 7.2%, considerably lower than the Saudi pooled rate. Likewise, studies in Turkey and India [25,26] found prevalence rates of approximately 9% and 11%, respectively, while Egypt and Ethiopia reported 21% and 26%, figures closer to the current findings [27,28]. The Pakistani multicenter survey revealed an even higher prevalence of 43%, though it included older children and broader diagnostic categories, which could explain the difference [29]. These variations likely reflect diverse diagnostic criteria, sampling methods, and cultural perceptions of bedwetting. Family history of enuresis emerged as a consistent risk factor across all international contexts. In Ethiopia, more than half of the affected children had mothers with a history of bedwetting [27], whereas in Turkey, 59% of enuretic children had an affected parent [25]. Similar patterns were evident in Saudi studies, where parental and sibling histories were among the most frequent predictors [19]. This underscores the strong genetic contribution to bladder control maturation. Male predominance was reported in studies from India, Turkey, and Ethiopia, whereas the present analysis detected no statistically significant gender difference, possibly due to greater social awareness and reporting among both parents in recent Saudi surveys [25-27].

Psychosocial stressors, including parental conflict, family separation, or academic pressure, have been highlighted globally and were also prominent in Saudi data [4]. For instance, Alshahrani *et al*. found stressful events and family changes as major correlates [20]. Comparable findings were noted in the Yemeni and Egyptian studies, where family disruption and relocation were linked to increased enuresis [28,30]. Sleep disorders such as snoring and difficulty awakening were significant in both the Saudi and Egyptian populations, emphasizing the interplay between sleep quality and bladder control mechanisms [31,32].

Marked heterogeneity (I^2^= 99.4%) was observed across studies, which is expected in epidemiological syntheses of behavioral and developmental conditions [33]. Several factors likely contributed to this variability. First, study settings and recruitment strategies varied widely, including school-based, community-based, and online surveys, each with different respondent biases. Second, diagnostic definitions were inconsistent; some studies applied strict ICCS or DSM-5 criteria, while others relied on parental perception or frequency-based definitions. Third, age ranges differed, with some studies including adolescents up to 16 years, thereby influencing prevalence since enuresis decreases with age. Fourth, socio-economic and cultural variations across Saudi regions may have influenced parental reporting and willingness to disclose the condition due to stigma [34]. Methodological quality, assessed by the Newcastle-Ottawa Scale, also differed among studies, which could contribute to statistical dispersion. Finally, the high prevalence in specific studies, such as Sherah *et al*. may represent localized environmental or methodological outliers that inflated pooled estimates.

The extreme heterogeneity observed in this meta-analysis is not unexpected in epidemiological syntheses of behavioral and developmental conditions. Variability in recruitment settings, diagnostic criteria, age ranges, and data collection methods likely contributed substantially to between-study differences. In particular, the study reporting an exceptionally high prevalence may reflect broader diagnostic thresholds, reliance on parent-reported online surveys, or sampling of higher-risk populations. These methodological differences underscore the need for standardized definitions and measurement tools in future research [35].

Despite its high prevalence and psychosocial impact, enuresis remains under-recognized and undertreated in Saudi Arabia, with many families relying on home remedies rather than seeking medical care. While early identification through school or primary healthcare settings may facilitate timely intervention, such approaches should be carefully implemented. Screening initiatives must be accompanied by parental education, counseling, and stigma-reduction strategies to avoid unintended consequences, such as increased blame or punitive responses toward affected children [36]. Supportive, non-punitive management approaches are essential to mitigating the emotional burden of the condition [36].

Overall, the findings highlight the need for increased awareness among parents, educators, and healthcare providers, alongside integrated, multidisciplinary care involving pediatric, mental health, and sleep specialists. At a national level, the development of standardized diagnostic criteria, improved professional training, and culturally sensitive education programs may enhance early recognition, promote appropriate care-seeking behavior, and reduce the psychosocial burden of nocturnal enuresis among Saudi children [37].

**Limitations:** this study has several limitations that should be considered when interpreting the findings. First, all included studies employed cross-sectional designs, which preclude causal inference between identified risk factors and nocturnal enuresis. Second, substantial heterogeneity was observed across prevalence estimates, reflecting wide variability in study settings, regional populations, age ranges, diagnostic definitions, and data collection methods, including school-based surveys, community sampling, and online parent-reported questionnaires. Although random-effects modeling and sensitivity analyses were performed, residual heterogeneity remained high, indicating that pooled prevalence estimates should be interpreted cautiously and viewed as indicative of a range rather than a single definitive value.

Third, one included study reported an unusually high prevalence, which substantially influenced the overall pooled estimate. Sensitivity analysis excluding this outlier resulted in a lower pooled prevalence, highlighting the influence of methodological differences and reinforcing the need for cautious interpretation. Fourth, reliance on parent- or self-reported data in several studies introduces the possibility of recall bias, social desirability bias, and under- or over-reporting, particularly given the social stigma associated with enuresis. Fifth, key variables, including severity and frequency of enuresis, differentiation between monosymptomatic and non-monosymptomatic forms, psychosocial impacts, and management strategies, were inconsistently reported, limiting the ability to perform more granular subgroup or dose-response analyses. Sixth, the pooled proportion of primary nocturnal enuresis was derived from only a small subset of studies, which restricts the precision and generalizability of this specific estimate. Finally, the review protocol was not prospectively registered, and the literature search was restricted to studies published in English, introducing potential reporting and language biases. Formal assessment of publication bias was not conducted due to the limited number of included studies, which may further affect the certainty of the pooled estimates. Despite these limitations, this systematic review provides the most comprehensive synthesis to date of nocturnal enuresis prevalence and associated factors among Saudi primary school-aged children.

## Conclusion

Nocturnal enuresis remains a common and clinically relevant condition among Saudi primary school-aged children. While the overall meta-analysis estimated a pooled prevalence of approximately 22%, sensitivity analysis excluding a methodological outlier yielded a lower estimate of about 14%, highlighting substantial between-study heterogeneity and indicating that prevalence should be interpreted as a range rather than a single definitive value. Primary nocturnal enuresis constitutes the majority of reported cases. Multiple factors were consistently associated with nocturnal enuresis, including positive familial history, psychosocial stressors, sleep disturbances, lower parental education, and comorbid conditions such as recurrent urinary tract infections and obesity. Beyond its physical manifestations, enuresis is associated with meaningful psychosocial consequences for affected children and their families, including embarrassment, emotional distress, variable parental responses, and potential impairment in school performance. Despite the availability of effective management strategies, such as behavioral interventions, enuresis alarms, and pharmacological treatment, a substantial proportion of affected children do not receive formal medical care. These findings underscore the need for improved awareness among parents, educators, and healthcare providers, alongside early identification and supportive, non-punitive intervention approaches. Integrating clinical management with parental education and school-based health initiatives may help reduce stigma, improve care-seeking behavior, and ultimately mitigate the psychosocial burden of nocturnal enuresis in this population.

### What is known about this topic

- Enuresis is one of the most common developmental disorders in childhood and is associated with psychosocial distress, embarrassment, and impaired academic performance;
- Global prevalence varies widely, with established risk factors such as younger age, male sex, family history, sleep disturbances, and psychosocial stress;
- In Saudi Arabia, several studies have assessed enuresis, but findings remain inconsistent, with large variability in reported prevalence and limited synthesis of associated determinants and psychosocial impacts.

### What this study adds

- This systematic review and meta-analysis provides the first pooled prevalence estimate of enuresis among Saudi primary school-aged children, identifying that approximately one in five is affected;
- It synthesizes evidence on key risk factors-including family history, psychosocial stress, sleep difficulties, urinary tract infections, and obesity-and highlights their consistency across studies;
- By summarizing the emotional, behavioral, and academic consequences of enuresis, this study underscores the need for early identification, greater parental and clinical awareness, and integrated school- and community-based interventions to reduce its burden.

## References

[1] Daley SF, Rincon MG, Leslie SW (2025). Enuresis. Pediatric Surgery: Pediatric Urology.

[2] Thurber S (2017). Childhood Enuresis: Current Diagnostic Formulations, Salient Findings, and Effective Treatment Modalities. Arch Psychiatr Nurs.

[3] Schaeffer AJ, Diamond DA (2014). Pediatric urinary incontinence: Classification, evaluation, and management. African Journal of Urology.

[4] Adisu MA, Habtie TE, Munie MA, Bizuayehu MA, Zemariam AB, Derso YA (2025). Global prevalence of nocturnal enuresis and associated factors among children and adolescents: a systematic review and meta-analysis. Child Adolesc Psychiatry Ment Health.

[5] Jalkut MW, Lerman SE, Churchill BM (2001). Enuresis. Pediatr Clin North Am.

[6] Nevéus T (2017). Pathogenesis of enuresis: Towards a new understanding. Int J Urol.

[7] Warne N, Heron J, von Gontard A, Joinson C (2024). Mental health problems, stressful life events and new-onset urinary incontinence in primary school-age children: a prospective cohort study. Eur Child Adolesc Psychiatry.

[8] Al-Zaben FN, Sehlo MG (2015). Punishment for bedwetting is associated with child depression and reduced quality of life. Child Abuse Negl.

[9] Birdal S, Dogangün B (2016). Behavioural problems in children with enuresis. Turk Pediatri Ars.

[10] Al-Zahrani S (2014). Nocturnal enuresis and its treatment among primary-school children in Taif, KSA. Int J Res Med Sci.

[11] Sherah KM, Elsharief MW, Barkat N, Jafery A (2019). Prevalence of nocturnal enuresis in school-age children in Saudi Arabia. Int J Med Develop Ctries.

[12] Almutairi NG, Alzahrani HM, Alhomrani MA, Alowid FK, Alghaith DM, Almutairi RH (2024). Prevalence of nocturnal enuresis among children and adults in Saudi Arabia: a systematic review and meta-analysis. Ann Saudi Med.

[13] Cumpston M, Li T, Page MJ, Chandler J, Welch VA, Higgins JP (2019). Updated guidance for trusted systematic reviews: a new edition of the Cochrane Handbook for Systematic Reviews of Interventions. Cochrane Database Syst Rev.

[14] Page MJ, McKenzie JE, Bossuyt PM, Boutron I, Hoffmann TC, Mulrow CD (2021). The PRISMA 2020 statement: an updated guideline for reporting systematic reviews. BMJ.

[15] Ouzzani M, Hammady H, Fedorowicz Z, Elmagarmid A (2016). Rayyan-a web and mobile app for systematic reviews. Syst Rev.

[16] Stang A (2010). Critical evaluation of the Newcastle-Ottawa scale for the assessment of the quality of nonrandomized studies in meta-analyses. Eur J Epidemiol.

[17] Lau J, Ioannidis JPA, Terrin N, Schmid CH, Olkin I (2006). The case of the misleading funnel plot. BMJ.

[18] Sterne JA, Sutton AJ, Ioannidis JP, Terrin N, Jones DR, Lau J (2011). Recommendations for examining and interpreting funnel plot asymmetry in meta-analyses of randomised controlled trials. BMJ.

[19] Alhejji MS (2021). Prevalence of Nocturnal Enuresis among Primary School Students in Jeddah-Saudi Arabia. ECNeurology.

[20] Alshahrani A, Selim M, Abbas M (2018). Prevalence of nocturnal enuresis among children in Primary Health Care Centers of Family and Community Medicine, PSMMC, Riyadh City, KSA. J Family Med Prim Care.

[21] Alnajjar M (2017). Prevalence and risk factors associated with nocturnal enuresis among primary school children in Al-Eskan Region, Makkah Al-Mokarramah. Int J Med Res Prof.

[22] Alshehri AA, Zaki MSH, Nour SO, Gadi WH, Zogel BA, Alfaifi SM (2023). Sleep-Disordered Breathing and Its Association with Nocturnal Enuresis at the Primary Schools in Saudi Arabia: A Cross-Sectional Study. Children (Basel).

[23] Shahin MM, Al-Shamary YW, Ahrashed LK, Al-Motiri SS, Alhazmi SM, Alswab WS (2017). The epidemiology and factors associated with nocturnal enuresis among school & preschool children in Hail City, Saudi Arabia: A cross-sectional study. Int J Adv Res.

[24] Ntais C, Talias MA (2024). Unveiling the value of meta-analysis in disease prevention and control: a comprehensive review. Medicina.

[25] Khadke DN, Dasila P, Kadam NN, Siddiqui MS (2023). Prevalence of nocturnal enuresis among children aged 05 to 10 years. Int J Contemp Pediatrics.

[26] Sarici H, Telli O, Ozgur BC, Demirbas A, Ozgur S, Karagoz MA (2016). Prevalence of nocturnal enuresis and its influence on quality of life in school-aged children. J Pediatr Urol.

[27] Ibrahim NH, Tolessa D, Mannekhulihe E (2021). Prevalence and Factors Associated with Enuresis among Children in Adama City, Oromia Regional State, Ethiopia. Int J Physiatry.

[28] Salem SA, Araby EM, Abd El Hie OM, Mohasseb MM, Abdo NM (2024). Prevalence and Quality of Life Among Children with Mono Symptomatic Nocturnal Enuresis at Benha City, Egypt. Benha Medical Journal.

[29] Shah S, Jafri RZ, Mobin K, Mirza R, Nanji K, Jahangir F (2018). Frequency and features of nocturnal enuresis in Pakistani children aged 5 to 16-years based on ICCS criteria: a multi-center cross-sectional study from Karachi, Pakistan. BMC Fam Pract.

[30] Al-Zubairi LM, Al-Emad AA, Mohanna M Bin, Al-Bada´ani TH (2018). Prevalence of Nocturnal Enuresis among Schoolchildren in Sana´a City, Yemen. Yemeni Journal for Medical Sciences.

[31] Baklola M, Terra M, Al-barqi M, AbdulHusain YH, Asiri SA, Jadaan NS (2024). Prevalence of insomnia among university students in Saudi Arabia: a systematic review and meta analysis. Egyptian Journal of Neurology. Psychiatry and Neurosurgery.

[32] Ge TJ, Vetter J, Lai HH (2017). Sleep Disturbance and Fatigue Are Associated With More Severe Urinary Incontinence and Overactive Bladder Symptoms. Urology.

[33] West SL, Gartlehner G, Mansfield AJ, Poole C, Tant E, Lenfestey N (2010). Comparative Effectiveness Review Methods: Clinical Heterogeneity. Rockville (MD): Agency for Healthcare Research and Quality (US).

[34] Alarfaj HM, Almaqhawi A, Kamal AH, Bu Bshait MS, Al Abdulqader A, Albarqi M (2024). Parental perception of nocturnal enuresis in a local region of Saudi Arabia. J Med Life.

[35] Glazener CM, Evans JH, Peto RE (2004). Complex behavioural and educational interventions for nocturnal enuresis in children. Cochrane Database Syst Rev.

[36] Yilmaz ES, Büyük ET (2021). Effect of education given to children with enuresis on quality of life. J Pediatr Urol.

[37] Sevecke JR, Meadows TJ (2018). It Takes a Village: Multidisciplinary Approach to Screening and Prevention of Pediatric Sleep Issues. Med Sci (Basel).

